# Women’s lived experiences of first-episode psychosis care: implications for gender-responsive care

**DOI:** 10.1192/bjo.2026.12085

**Published:** 2026-09-01

**Authors:** Grainne McGinty, Sean Naughton, Keith Gaynor, Roisin Farrelly, Philip O’Donnell, Anja Stano, Mary Clarke

**Affiliations:** Dublin and East Treatment and Early Care Team (DETECT), Dublin, Ireland; School of Medicine, RCSI University of Medicine and Health Sciences, Dublin, Ireland; Psychosis Research Centre, St John of God Hospitaller Services Group, Dublin, Ireland; School of Psychology, University College Dublin, Ireland; Centre for Environmental Neuroscience, Max Planck Institute for Human Development, Berlin, Germany; School of Medicine, University College Dublin, Ireland

**Keywords:** First-episode psychosis, women’s mental health, gender-responsive care, early intervention in psychosis, qualitative research

## Abstract

**Background:**

Women with first-episode psychosis (FEP) may experience distinct clinical, reproductive and social challenges, yet their experiences of psychosis care remain underexplored. Greater understanding of women’s perspectives is needed to inform gender-responsive service provision.

**Aims:**

To explore how women make sense of their experience of psychosis care, to identify areas where gender-specific needs were unmet and to highlight priorities for improving care for women experiencing FEP.

**Method:**

This qualitative study employed semi-structured interviews with women who had experienced FEP and received care through early intervention in psychosis and adult community mental health services (*N* = 15). Data were analysed using reflexive thematic analysis.

**Results:**

Four overarching themes were identified: (a) violations of personhood and bodily integrity; (b) invisible menstrual and reproductive health in mental healthcare; (c) women-specific barriers to accessing and sustaining care; and (d) moving towards better care for women with FEP. Women described feeling unsafe in mixed-gender in-patient settings, experiencing unmet reproductive and menstrual health needs and navigating caregiving responsibilities that complicated engagement with care. Findings informed a framework of recommendations for improving gender-responsive psychosis care.

**Conclusions:**

This study provides one of the first in-depth qualitative examinations of women’s experiences of FEP care. Women described how their experiences were shaped by issues of safety, autonomy, reproductive health and gendered expectations that remain insufficiently recognised within current service models. These findings highlight persistent gaps in sex- and gender-responsive psychosis care, and support the development of services that more effectively address women’s safety, reproductive health and caregiving needs.

Gender disparities exist in the clinical presentation, risk factors, prevalence and outcomes of psychosis,^1,2^ reflecting patterns increasingly recognised across other areas of healthcare, including cardiovascular medicine.^3^ However, despite clear evidence of gender differences, clinical services continue to adopt gender-neutral approaches that risk overlooking the unique needs of women.^4,5^ Whereas our focus is on cis-gendered women, who comprise the large majority of those attending Early Intervention in Psychosis (EIP) services, we recognise that sex and gender exist along continua and intersect in ways that extend beyond binary categories, and clinical services must be responsive to these complexities.

Women tend to present with first-episode psychosis (FEP) later than men, often around key hormonal or reproductive transitions such as the menstrual cycle, postpartum and perimenopause. These periods have been associated with increased vulnerability to symptom emergence or relapse.^6,7^ Such patterns highlight a notable interaction between reproductive health and psychosis that is rarely accounted for in routine psychiatric care.

Women also often display different early symptom profiles, typically fewer negative symptoms and less cognitive impairment, but more prominent affective and trauma-related symptoms and have lower rates of substance misuse.^8,9^ Such presentations can contribute to diagnostic overshadowing, with clinicians interpreting early psychotic symptoms through affective or psychosocial lenses, leading to delays in recognition and referral to early intervention services.^10–12^ Together, these gendered patterns illustrate how ostensibly gender-neutral service models risk overlooking the specific ways in which psychosis emerges and is identified in women.

More recent longitudinal research challenges earlier assumptions of a more favourable course of illness in women. Although women often present later and with fewer risk factors, they experience similar or higher rates of relapse, rehospitalisation and persistent affective symptoms compared with men.^13–15^ Recent reviews suggest that these disadvantages may not reflect inherent illness trajectories, but rather suboptimal care, informed by research excluding women, and therefore failure to recognise their distinct needs.^2^

Several structural and clinical factors contribute to this gap. Women remain underrepresented in some areas of psychiatric research, and sex- and gender-specific analyses are frequently absent even when women are included. As a result, the evidence base informing psychiatric care may not adequately capture women’s experiences, treatment responses or reproductive health needs.^16^ As a result, services often pay inadequate attention to reproductive and menstrual health needs; in addition to caregiving responsibilities, stigma surrounding motherhood and fears of child welfare intervention can delay help-seeking and complicate engagement with services.^17,18^ Trauma-informed practices are also insufficiently integrated into many services, despite the high prevalence of trauma among women with psychosis.^19–21^ Furthermore, upper age thresholds of 35 years in many EIP services disproportionately exclude women who present later in life.^22^ Recent calls have argued that EIP services should incorporate gender-specific and lifespan-informed approaches to care.^23^

Whereas clinician perspectives on gender-sensitive EIP provision have recently gained attention,^24^ there remains a lack of research exploring the lived experiences of women navigating these services, prompting calls for qualitative work that centres women’s voices to inform more gender-responsive early intervention models.^11,25^

The present study addresses this gap by qualitatively exploring the experiences of cis-gender women accessing EIP services in Ireland. Through reflexive thematic analysis, we examine how women make sense of their experience of psychosis and highlight priorities for improving care for women experiencing FEP across in-patient, community and early intervention settings.

## Method

### Study design

This qualitative study employed semi-structured interviews with women who had experienced FEP and received care through an EIP service. This Irish service operates a hub-and-spoke model in which specialist EIP interventions are delivered in partnership with local community mental health teams (CMHTs) through a shared-care approach. Consequently, participants reflected on their experiences across the range of services involved in their care, including EIP services, CMHTs and, where relevant, in-patient psychiatric care.

The study adopted an inductive, constructivist epistemology, recognising that knowledge is co-constructed through the interaction of participants’ narratives, contextual factors and researcher interpretation. Reflexive thematic analysis (RTA)^26^ guided data analysis, enabling flexible engagement with the data while foregrounding reflexivity. RTA was chosen because it supports in-depth exploration of lived experience and allows nuanced attention to the gendered and contextual dimensions of women’s interactions with EIP services.

Topic guide questions were co-developed following a review of the literature on women’s experiences of first-episode psychosis by the research team (G.M., M.C., S.N. and K.G.), and in collaboration with two individuals with lived experience. Their input helped refine the interview guide and ensure that it addressed topics considered meaningful and relevant by women with lived experience of first-episode psychosis (see Table 1). Guided by a constructivist epistemology, interviews focused on how participants made meaning of their experiences of receiving psychiatric care for psychosis. Interviews were understood as co-constructed encounters in which meaning was shaped through dialogue between participant and researcher. Reflexive diary notes documented the researcher’s assumptions, emotional responses and evolving interpretations, and these reflections informed ongoing questioning and analytic decision-making. A researcher positionality statement is provided in the supplementary materials available at https://doi.org/10.1192/bjo.2026.12085.

**Table 1:** Interview schedule Table 1 long description.

Section	Topic area	Main question
Section 1	Barriers to care	As a woman, did you face any challenges when trying to get help or support for your mental health?
	Engagement with services	After your first contact, did you find it easy or difficult to stay engaged with the service?
	Improving your care	Looking back, is there anything that could have improved the care you received as a woman or service user?
Section 2	Hormonal and reproductive health	Were menstruation, pregnancy, fertility or menopause discussed during your care?
	Motherhood and caring roles	If you are a mother, did the service recognise your role when supporting your mental health?
Section 3	Information and support	Did the information you received feel relevant and meaningful to you as a woman?
	Medication considerations	Were medication explanations clear and easy to understand?
	Physical health support	Did the service consider your physical health alongside your mental health?
Section 4	Psychological and social support	Did you receive emotional or social support that felt appropriate for you as a woman?
	Impact of being a woman	Do you think your experience of care was influenced by being a woman?
Section 5	Future improvements	What changes could improve mental health services for women?

### Participants and procedure

Participants were recruited from two community mental health services in Ireland that had access to an EIP service. A purposive sampling strategy was employed, with consultant psychiatrists and multidisciplinary team clinicians identifying suitable candidates who met the inclusion criteria of self-identifying as female and being clinically stable with regard to participation.

Maximum-variation sampling was used to capture heterogeneity across characteristics theoretically relevant to women’s experiences of psychosis care, including age, diagnostic profile, caregiving and parental status, stage of illness and level and type of mental health service engagement.

Fifteen women aged between 19 and 65 years participated (*N* = 15). All had experienced a first episode of psychosis and had received care through EIP and/or adult CMHT. Participants were recruited on the basis of self-identifying as women. Gender identity was not formally assessed as part of the study, and the sample available for recruitment did not include any individuals known to identify as transgender or gender diverse. Participant demographic characteristics (including age, parental status and employment status) are presented in Table 2.

**Table 2:** Participant demographics Table 2 long description.

Demographics	*n* (%)
Age, years	15 (100)
18–24	1 (6.6)
25–34	2 (13.3)
35–44	4 (26.7)
45–54	6 (40)
55–65	2 (13.3)
65+	0 (0)
Parental status
Children	6 (40)
No children	9 (60)
Employment
Employed (part-/full-time)	6 (40)
Unemployed	4 (26.7)
Illness leave	1 (6.6)
Retired	1 (6.6)
Student	3 (20)

Participants were initially approached via telephone by a clinical gatekeeper, provided with an information sheet and contacted by the research team only if they expressed interest and consented to being contacted. Written informed consent was obtained prior to interviews. Interviews lasted between 45 and 90 min and followed a topic guide exploring the following: experiences of safety and trust in services; the role of reproductive and hormonal health in care; service accessibility; motherhood and caregiving responsibilities; clinical encounters and communication; experiences of medication; and preferences for support as a woman.

### Data analysis

Interviews were all conducted remotely via telephone in a private room, audio-recorded, transcribed verbatim and anonymised. Analysis followed Braun and Clarke’s six phases of RTA.^27^ Initial coding was conducted in Microsoft Excel (Microsoft 365; Microsoft Corporation, Redmond, Washington, USA; Windows 11; https://www.microsoft.com/microsoft-365/excel), using free-text and colour-coding by G.M. and R.F. Codes were then collated into themes, refined through iterative team discussion and finalised into four overarching themes. Reflexivity was central to the process, with memo-writing used to document assumptions and interpretative decision-making. Two researchers independently coded transcripts and discussed discrepancies with the research team, to enhance rigour.

## Results

A reflexive thematic analysis of interviews with 15 cis-gender women attending an EIP service in Ireland identified four overarching themes: (a) violations of personhood and bodily integrity; (b) invisible menstrual and reproductive health in mental healthcare; (c) women-specific barriers to accessing and sustaining care; and (d) moving towards better care for women with FEP (see Table 3 for supporting quotations).

**Table 3:** Themes, subthemes and exemplar quotations Table 3 long description.

Theme	Subtheme	Exemplar quotation
Theme 1. Violations of personhood and bodily integrity	Sexual risk and fear in care settings	‘Well, the ward was a unisex ward, and I felt I was being intimidated by a male patient. Sexually intimidated, actually… I didn’t get any support or feel supported by the staff… I don’t think I was taken seriously actually.’ (Participant 6 (P6))
	Dehumanisation and loss of autonomy	‘There was no consideration for the fact that I was in menopause… I asked for cotton sheets. I wanted to bring in my own sheets, I wasn’t allowed.’ (P6)
Theme 2. Invisible menstrual and reproductive health in mental healthcare	Menopause	‘If you’re in menopause, it doesn’t make much difference. It was never discussed in my care.’ (P11)
	Menstrual cycle and symptoms	‘Only recently I tracked back and remembered that when the thing happened that triggered all this kind of stuff… I actually had my period around that time, and I only remembered that recently.’ (P4)
	Reproductive health and fertility	‘They said if I needed medication for that, they’d prescribe it. But I feared infertility, and nobody explained it properly. I’d like children and I wonder if it’s to do with hormone imbalance that I’ve had no luck.’ (P10)
Theme 3. Women-specific barriers to accessing and sustaining care	Childcare and caregiving responsibilities	‘It’s a very ugly, scary process… I could have told the kids and packed a bag. Instead, I disappeared during dinner. There should be more family involvement at the early stage.’ (P5)
	Feeling invalidated and voice unheard	‘They might underestimate me a little bit, thinking I couldn’t cope. It felt more judgemental than supportive.’ (P12)
	Unfulfilled needs for practical support	‘Helping, making sure you have support and getting the baby registered and getting them into creches… making sure that you don’t need support doing all that and staying mentally well.’ (P12)
	Side-effects of medication	‘I realised then that wasn’t explained often enough… the weight gain was the biggest problem I had… Smaller portions or portion control would be a great help.’ (P13)
Theme 4. Moving towards better care for women with first-episode psychosis	Preferences for women-only spaces, especially in-patient	‘It would have made a huge difference if it had been women… when you’re at your lowest… as a woman, I was vulnerable like that.’ (P8)
	Preference for female clinicians	‘Perhaps… it would have been easier maybe to talk to a woman psychiatrist just because of the nature of my problems.’ (P7)
	Communication, information and joined-up care	‘Maybe linking in with the GP so they share knowledge. Joined-up thinking would help.’ (P8)
	Continuity and trust in clinician relationships	‘Staying with the same person is important… it’s very hard to open up if they change every 6 months.’ (P10)
	Practical improvements suggested	‘Maybe even just one session later in the evening… but it’s very much a nine-to-five service.’ (P8)

GP, general practitioner.

### Theme 1: violations of personhood and bodily integrity

This theme reflects women’s accounts of feeling unsafe, intimidated or overlooked in in-patient psychiatric environments, all of which happened to be mixed-gender wards. Women described feeling unsafe, disbelieved and stripped of agency. Whereas participants reflected on care received across multiple service settings, concerns relating to physical and sexual safety were discussed primarily in the context of in-patient admissions. Two subthemes were identified: (a) sexual risk and fear in care settings and (b) dehumanisation and loss of autonomy.

#### Sexual risk and fear in care settings

Many participants recounted experiences of sexual intimidation or threat during in-patient stays. Several described their experiences on mixed-gender wards as profoundly unsafe:

‘I didn’t feel safe on the ward with men… One said he was going to knife me. I was terrified.’ (Participant 5 (P5))

Women linked this lack of safety to systemic gender-blindness in in-patient provision, notably the lack of single-gender wards, which necessitated mixed-gender ward placement:

*‘*It was a mixed-sex ward, and I felt sexually intimidated. I wanted to call the police.’ (P6)

‘Even when I wasn’t directly threatened, I was always on edge. You never relaxed, you’re constantly alert.’ (P9)

‘In hospital there was a man who was kind of saying like he kind of wanted me as a girlfriend and all that stuff. But the hosp – but then the nurses were like… very like supportive.’ (P4)

Some accounts illustrated how some women felt that their safety concerns were not fully acknowledged by the service, which contributed to feelings of vulnerability.

#### Dehumanisation and loss of autonomy

Beyond physical safety, participants expressed feelings of humiliation and that female-specific needs were not addressed:

*‘*I felt sorry for people that might be menstruating, actually, because the toilets were often unusable. And that’s again, back to the men and the women in one, often the men would use the women’s toilets, which was really frustrating, and then leave them in a mess.’ (P6)

‘There was no consideration for the fact that I was in menopause, no acknowledgement of the things I needed to keep myself comfortable. I was sleeping in a bed with almost what felt like plastic sheets, and I was sweating like crazy and… I asked for cotton sheets. I wanted to bring in my own sheets, I wasn’t allowed.’ (P6)

These accounts suggested that participants’ experiences of dignity and autonomy extended beyond clinical care and were influenced by whether everyday women-specific needs, such as privacy, menstruation and menopausal symptoms, were adequately accommodated within the in-patient environment.

### Theme 2: invisible menstrual and reproductive health in mental healthcare

Participants highlighted how hormonal, reproductive and gynaecological issues were seldom discussed or integrated into their mental healthcare. Three interlinked subthemes emerged: (a) menopause and perimenopausal experiences; (b) menstrual cycle and symptom fluctuation; and (c) reproductive health and fertility concerns.

#### Menopause and perimenopausal experiences

Women reported that menopausal symptoms were seldom acknowledged within their care, even when these affected mood:

*‘*If you’re in menopause, it doesn’t make much difference. It was never discussed in my care.’ (P11)

[Re. menopause] ‘But there will, when I go into it there will have to be a conversation, but I think I’ll be the one bringing it up.’ (P5)

Several described the intersection between menopausal transition and psychotic vulnerability yet found no structured support for this phase: ‘No, I didn’t have any effects yet, just started into menopause, but it was never discussed.’ (P8)

#### Menstrual cycle and symptom fluctuation

Participants frequently linked mood or psychotic symptom exacerbation to their menstrual cycles:

*‘* I notice I’m more anxious and drained during my period, it definitely affects my recovery.’ (P8)

‘Only recently I tracked back and remembered that when the thing happened that triggered all this kind of stuff – last spring, whether you want to call that psychosis or – or a spiritual awakening, or a seizure or whatever. Em, I actually had my period around that time, and I only remembered that recently.’ (P4)

#### Reproductive health and fertility concerns

Concerns about fertility, prolactin-related effects and medication risks were described as insufficiently addressed:

*‘*I feared infertility, and nobody explained it properly… I’d like children and I wonder if it’s to do with hormone imbalance that I’ve had no luck.’ (P10)

Others described prolonged amenorrhoea and felt that this was not closely followed up in their care:

*‘*I wasn’t ready to have a baby, but it was upsetting. I felt like my womanhood was taken away.’ (P12)

‘But no period for like 8 years or something. That’s like prime age for having children.’ (P13)

### Theme 3: women-specific barriers to accessing and sustaining care

This theme encompassed structural, relational and social barriers that uniquely hindered women’s engagement with EIP services. Three subthemes were identified: (a) childcare and caregiving responsibilities; (b) feeling invalidated and unheard; and (c) medication side-effects and embodiment.

#### Childcare and caregiving responsibilities

Motherhood shaped women’s ability to seek and sustain care. Sudden admissions were described as traumatic for both mothers and children:

*‘*I disappeared during dinner; my kids had no idea where I’d gone.’ (P5)

Some described practical inflexibilities:

‘There should be out-of-hours support… especially for women with postpartum depression or childcare duties.’ (P12)

#### Feeling invalidated and unheard

Women reported feeling dismissed or invalidated within clinical interactions:

‘They might underestimate me a little bit, thinking I couldn’t cope. It felt more judgemental than supportive.’ (P12)

Past traumatic experiences were sometimes experienced as being met with insufficient acknowledgement or understanding:

*‘*He didn’t believe me… he was telling everything back to my actual abuser… it made me extremely fearful.’ (P10)

#### Medication side-effects and embodiment

Weight gain, hormonal changes and sexual side-effects significantly affected well-being and adherence:

*‘*I put three stone on from the olanzapine… a bit of information… would definitely have helped.’ (P11)

Sexual side-effects were also distressing:

*‘*It really affected me. It killed my sex drive.’ (P13)

### Theme 4: moving towards better care for women with FEP

The final theme captured women’s visions for safer, more responsive and relationally attuned care. Subthemes included the following: (a) preference for female clinicians and women-only spaces; (b) improved communication and information-sharing; and (c) practical recommendations for service reform.

#### Preference for female clinicians and women-only spaces

Many participants expressed greater comfort with female staff and advocated for women-only wards or groups:

*‘*When you’re at your lowest, being around men can feel unsafe. A women-only ward would make a huge difference.’ (P8)

‘I’ve two female doctors. So, you know, I think that’s helpful… Yeah, I don’t think I would have had felt as supported by male doctors.’ (P6)

‘I had some really embarrassing bodily function-related problems, I couldn’t articulate certain things the way I would have articulated if I talked to a woman.’ (P7)

Participants also noted that it would be helpful to have a choice of male or female clinicians:

*‘*Maybe like if you’re gonna see your psychiatrist or psychologist, they… ask like which gender you prefer.’ (P3)

#### Improved communication and information-sharing

Participants emphasised the need for transparent, coordinated information across providers:

*‘*If psychiatry and GP [general practitioner] actually talked, I wouldn’t have to explain my story again every time.’ (P8)

Women also sought clearer guidance on medications, physical health and available supports:

*‘*Definitely more information, more support would help.’ (P12)

Building consistent therapeutic relationships was central to recovery:

*‘*Staying with the same person is important… it’s very hard to open up if they change every 6 months.’ (P10)

#### Practical recommendations for service reform

Participants proposed tangible service adaptations, including flexible scheduling and tailored medication planning:

*‘*Maybe even just one session later in the evening… it’s very much a nine-to-five service.’ (P8)

Others emphasised shared decision-making:

*‘*In future I’d like to try to up the medication first… and then see if hospital is needed.’ (P5)

## Discussion

This study provides one of the first in-depth qualitative examinations of women’s experiences of FEP within EIP and adult mental health services in Ireland. The findings reveal that women described how their experiences of care were shaped profoundly by issues of safety, autonomy and gendered expectations, many of which remain insufficiently recognised in current service design. Across participants’ accounts, women described feeling unsafe in mixed-gender in-patient settings, a perception that hormonal and reproductive health needs were not well addressed and being overburdened by caregiving responsibilities. Collectively, these findings highlight persistent gaps in sex- and gender-responsive psychosis care, and reinforce calls within the international literature to better incorporate women’s lived experiences into service development.^11,25^

### Women’s safety, autonomy and trust: a fundamental gap in in-patient psychiatric environments

Women’s accounts consistently foregrounded physical and sexual safety as important aspects of their care experience, with these concerns arising primarily in relation to in-patient admissions rather than community-based EIP or general adult services. Mixed-gender in-patient wards were described as unpredictable and, for many, frightening environments marked by intimidation, sexual harassment and inadequate awareness of women’s needs and safety concerns. These findings echo longstanding concerns about gender-based violence in psychiatric settings^18,28^ and align with recent critiques that FEP care insufficiently addresses women’s safety needs, particularly at admission and during acute distress.^29^

Experiences that participants interpreted as being ignored, isolated or lacking autonomy further undermined their sense of trust and safety. These dynamics reflected broader patterns of gendered disempowerment in acute psychiatry, where women are more likely to report feeling dismissed, infantilised or disbelieved.^16^ The present findings reinforce the need for trauma-informed, gender-sensitive in-patient policies and the expansion of women-only spaces as a matter of basic safety and dignity.

### Invisible menstrual and hormonal health: the neglected clinical domain

Across the sample, participants described an absence of attention to menstrual, perimenopausal and reproductive health, despite the potential relevance of hormonal transitions in the exacerbation of psychosis symptoms.^5^ Women reported prolonged amenorrhoea, prolactin-related symptoms, menopausal discomfort or changes in symptom severity around their cycle, and several felt that these concerns were not fully reflected in their treatment discussions. Recent clinical guidance has also highlighted the importance of considering sex-specific factors when selecting antipsychotic medication for women with FEP, including reproductive health, endocrine effects and differential side-effect profiles.^30^

This disconnect mirrors critiques that psychiatric care remains ‘gender-blind’ in relation to women’s physiology.^31^ Similar sex- and gender-based biases have been identified in other areas of medicine, including cardiac care, where research underpinning clinical guidelines has historically underrepresented women’s experiences and presentations.^3^ Failure to inquire about or accommodate menstrual experiences not only compromises clinical understanding but contributes to women feeling unseen, misunderstood and physically uncomfortable within services. For women experiencing psychosis, especially during high-risk phases such as postpartum or perimenopause, this omission represents a substantive clinical gap with potentially serious consequences.

### Gendered barriers to access and care continuity

The women in this study described caregiving responsibilities, fear of judgement as mothers and a lack of flexible service provision as major obstacles to care. Sudden in-patient admissions were particularly distressing, often occurring without opportunity to prepare children or arrange support. These findings align with evidence that motherhood shapes help-seeking behaviour, and that stigma surrounding mental illness and parenting disproportionately affects women.^32^

Women also described feeling invalidated or unheard. Such experiences are known to erode engagement in mental health services, particularly for those with histories of interpersonal trauma or gender-based violence.^21,33^

### Towards gender-responsive EIP: implications for service development

Participants provided clear and actionable recommendations for improving women’s experiences of FEP care. These priorities are consistent with emerging frameworks for sex- and gender-sensitive psychosis care, which argue that gender-neutral EIP models overlook foundational aspects of women’s safety, embodiment and social context.^25^ Women’s recommendations therefore reflect not only personal preference but also systemic shortcomings in service design.

To support translation into practice, the study’s findings can be distilled into a set of concrete clinical recommendations, presented below.

- Sex- and gender-sensitive safety in in-patient environments. Women described feeling unsafe in mixed-gender in-patient settings, highlighting the need for access to women-only beds or clearly designated female spaces. Whereas Irish in-patient services operate as approved centres with mandatory policies on safety, privacy and dignity, women’s accounts suggest that the consistency and effectiveness of these measures in mixed-gender settings require further attention. Broader consultation on this matter with women who have lived experience may be useful to explore this further. Ensuring physically and psychologically safe environments is foundational to therapeutic engagement and recovery, both locally and internationally.
- Integrating reproductive, menstrual and hormonal health. Routine psychiatric care should incorporate enquiry about menstrual cycles, hormonal transitions (including postpartum and perimenopause) and reproductive health concerns. Monitoring the endocrine side-effects of antipsychotic medication should be standard practice, as should discussions regarding medication-related sexual side-effects, given their potential impact on well-being, relationships and treatment adherence. Women also highlighted the importance of receiving information regarding potential medication side-effects, including weight gain, and strategies to mitigate their impact. Enhanced integration across primary care, including general practitioners and menopause specialists, secondary care services and sexual and reproductive medicine and perinatal mental health services, would facilitate more holistic and coordinated care while reducing service fragmentation.
- Flexible and family-inclusive service delivery. Caregiving responsibilities were a significant barrier to accessing and sustaining care. Services should offer greater flexibility through adaptable appointment scheduling, home visits where appropriate and improved access to social work support to assist with childcare arrangements before and during admission.
- Trauma-informed, gender-responsive training. Training across both out- and in-patient settings should explicitly address hormonal transitions and the impacts of gender-based violence and trauma on women’s mental health. Although services may aim to operate within trauma-informed frameworks, women’s experiences suggest that consistency across care settings can vary, particularly regarding in-patient admissions. Building staff capacity to maintain trauma-informed, gender-responsive practice throughout care transitions is essential.
- Collaborative and integrated care pathways. Women emphasised the need for more joined-up care. Improved communication and coordination across mental, physical and reproductive health services would reduce fragmentation, ensure continuity and prevent important clinical information from being overlooked.

### Strengths and limitations

A major strength of this study is its exclusive focus on women with lived experience of FEP, an underrepresented group in psychosis research. The interviews explored a broad range of aspects of care provision, including in- and out-patient care and interventions. The use of reflexive thematic analysis allowed for nuanced, participant-centred interpretation.

Limitations include the absence of transgender participants, reflecting the demographic profile of the clinical population at the time of recruitment. Findings are also drawn from a single geographical area and may not fully reflect experiences across other healthcare services in Ireland or internationally. We are unable to provide detailed information regarding age at first episode, illness duration or previous hospitalisation history, limiting our ability to contextualise participants’ stage of recovery. Nor did we collect ethnicity data, limiting consideration of how this may have shaped participants’ experiences. Although there is a growing body of qualitative research examining women’s experiences of psychosis and early intervention, this remains a relatively underexplored area. As such, these findings should be interpreted cautiously. Nonetheless, the themes align with existing international literature and contribute to ongoing discussions about sex, gender and women’s experiences within EIP care.

### Summary

This study illuminates critical gaps in how women experience early psychosis care, revealing concerns about reproductive health awareness and gender-responsive in-patient care and practice. Women’s accounts suggest that current services are not yet fully equipped to meet their needs. This situation must be understood within the context of notable resource and funding constraints affecting mental health services more broadly. Embedding sex- and gender-sensitive, trauma-informed and hormonal understanding within psychosis care is essential to advancing equity, and is likely to lead to improved outcomes for women experiencing FEP.

## Supporting information

10.1192/bjo.2026.12085.sm001 McGinty et al. supplementary material McGinty et al. supplementary material

## Data Availability

The data that support the findings of this study are available from the corresponding author upon reasonable request.

## Supplementary material

The supplementary material is available online at https://doi.org/10.1192/bjo.2026.12085

## Table 1 Long description

A table with five sections and multiple topic areas, each with a main question. Section 1 focuses on barriers to care, engagement with services, and improving care, with questions about challenges faced, ease of staying engaged, and potential improvements. Section 2 covers hormonal and reproductive health, motherhood and caring roles, with questions about discussions on menstruation, pregnancy, fertility, menopause, and recognition of motherhood roles. Section 3 addresses information and support, medication considerations, and physical health support, asking about the relevance of information, clarity of medication explanations, and consideration of physical health. Section 4 explores psychological and social support and the impact of being a woman, with questions about emotional or social support and the influence of being a woman on the experience of care. Section 5 discusses future improvements, asking what changes could improve mental health services for women.

Navigate back to Table 1.

## Table 2 Long description

A table titled ‘Participant demographics’ with two columns: Demographics and n (%). The table has 15 rows, including a header row. The Demographics column lists age groups, parental status, and employment status. The n (%) column lists the number and percentage of participants in each category. Age groups are 18-24, 25-34, 35-44, 45-54, 55-65, and 65+. Parental status is divided into Children and No children. Employment status includes Employed (part-/full-time), Unemployed, Illness leave, Retired, and Student. The table shows that the highest percentage of participants (40 percent) are aged 45-54, 60 percent have no children, and 40 percent are employed either part-time or full-time.

Navigate back to Table 2.

## Table 3 Long description

The table presents a reflexive thematic analysis of interviews with 15 cis-gender women attending an early intervention psychosis service in Ireland. It is organized into four themes, each with subthemes and exemplar quotations. The table has four columns: Theme, Subtheme, and Exemplar quotation. Theme 1, Violations of personhood and bodily integrity, includes two subthemes: Sexual risk and fear in care settings and Dehumanisation and loss of autonomy. Theme 2, Invisible hormonal and reproductive health in mental healthcare, includes three subthemes: Menopause, Menstrual cycle and symptoms, and Reproductive health and fertility. Theme 3, Women-specific barriers to accessing and sustaining care, includes four subthemes: Childcare and caregiving responsibilities, Feeling invalidated and voice unheard, Unfulfilled needs for practical support, and Side-effects of medication. Theme 4, Moving towards better care for women with first-episode psychosis, includes five subthemes: Preferences for women-only spaces, especially in-patient, Preference for female clinicians, Communication, information and joined-up care, Continuity and trust in clinician relationships, and Practical improvements suggested. Each subtheme is accompanied by exemplar quotations that illustrate the themes and subthemes.

Navigate back to Table 3.
