# Supplementary material for: Women’s lived experiences of first-episode psychosis care: implications for gender-responsive care
Source: BJPsych Open. 2026 Sep 1;12(5):e225. doi: 10.1192/bjo.2026.12085 (PMC13748676; doi:10.1192/bjo.2026.12085)
Supplement: Supplementary file 1 [file S2056472426120857sup001.docx]

Researcher Positionality Statement

The researcher who conducted the interviews and led the analysis (GM) is a 30-year-old woman with lived experience of chronic illness, autoimmune disease, and long-term physical health challenges arising from sports-related injuries. These experiences have provided her with personal insight into navigating healthcare systems, managing ongoing health conditions, and encountering the ways in which women's physical symptoms and healthcare needs can be overlooked or insufficiently addressed. While these experiences are distinct from the experiences of psychosis described by participants, they contributed to a heightened awareness of issues relating to bodily autonomy, healthcare access, gendered experiences of care, and the impact of illness on identity and daily life.

GM approached the study from a position that women's experiences of healthcare are often shaped by broader social and structural factors, including gender norms and assumptions within healthcare systems. Throughout data collection and analysis, reflexive practices were used to critically examine how these perspectives may have influenced interpretation. Reflexive diary notes were maintained throughout the research process to document assumptions, emotional responses, and emerging interpretations. Regular discussions with the wider multidisciplinary research team provided opportunities to challenge individual perspectives and ensure that themes remained grounded in participants' accounts.

The research team comprised clinicians and researchers with backgrounds in psychiatry, psychology, and early intervention in psychosis. The team's diverse professional perspectives supported critical reflection throughout the analytic process and contributed to the development of themes that sought to faithfully represent participants' experiences while acknowledging the interpretative nature of qualitative research.
